# Associations of multisite pain with healthcare utilization, sickness absence and restrictions at work

**DOI:** 10.1007/s00420-016-1141-7

**Published:** 2016-05-12

**Authors:** Rita Cássia Pereira de Fernandes, Alex Burdorf

**Affiliations:** 1 Department of Social and Preventive Medicine, School of Medicine, FMB, Federal University of Bahia, Largo do Terreiro de Jesus, s/n. Centro Histórico, Salvador, Bahia 40026-010 Brazil; 2 Department of Public Health, Erasmus MC, University Medical Center Rotterdam, P.O. Box 2040, 3000 CA Rotterdam, The Netherlands

**Keywords:** Multisite pain, Sickness absence, Widespread pain, Work disability, Musculoskeletal pain, Healthcare utilization

## Abstract

**Purpose:**

Despite the apparent importance of multisite musculoskeletal pain (MMP) for functioning, there is still a lack of studies that have investigated the influence of MMP on healthcare utilization (HU), sickness absence (SA) and restrictions of work (RW). This study described the HU, SA and RW due to musculoskeletal pain (MP) in different body sites and according to number of pain sites and investigated associations between number of pain sites with these three outcomes in workers from Bahia, Brazil.

**Methods:**

This study was based on two cross-sectional surveys carried out in 2010 and 2012. The response in the pooled data was 97 % (*n* = 1070, 228 women and 842 men). Interviewer-administered questionnaire was used with questions on HU, SA and RW due to MP. The number of pain sites is the sum score of eight body sites with pain in previous 12 months. Covariates were age, gender, physical and psychosocial work demands, leisure-time physical activities and body mass index. Cox regression models, properly applied to a cross-sectional study, determined the associations between number of pain sites with the three outcomes.

**Results:**

Prevalence of MP in the previous 12 months is 81.2 %, and MMP accounted for two-thirds of pain. We found consistently increasing occurrence of HU, SA and RW with increasing number of pain sites. For individuals with pain in four or more body sites, the utilization of health care was 1.7-fold the utilization by workers with single-site pain. Having pain in four or more sites increased the prevalence of SA 3.6-fold and of RW 4.0-fold compared with having single-site pain, after adjustment by covariates.

**Conclusions:**

The functional consequences of pain depend on how much body regions are affected, i.e., the more widespread pain, the higher the likelihood of medical consumption, sickness absence and restricted work. Given the high comorbidity, the number of pain sites, instead of specific body site of pain, seems to be a useful measure to anticipate interventions at workplaces for musculoskeletal disease prevention.

## Background

Musculoskeletal pain is a relevant health problem in industrialized and developing countries. It is an important cause of productivity loss at work and a burden to the social security system due to its contribution to sickness absence and work-related disability. Musculoskeletal pain causes human suffering, especially to workers, who have to perform their tasks under the very same work conditions possibly related to the causality of their health problem.

The National Brazilian Social Insurance System has ratified musculoskeletal diseases (MSDs) as the most frequent occupational disease in recent years. In 2012, MSDs accounted for 84 % of all diagnoses among the 50 most prevalent occupational diseases (Brasil 2014). It is noteworthy that this information from the Brazilian Social Insurance System applies to the population with formal employment relationship, and refers to workers who presented clinical symptoms of such severity that they were attributed a temporary work disability benefit.

Souza and Santana (2011) showed that the incidence of a work-related disability benefit due to MSDs in the neck and upper limbs was 14.6 cases per 10,000 workers in 2008, in the city of Salvador, Brazil. In the USA, the reported incidence was 3.2 cases per 10,000 in the same year. Since in Brazil, the social benefits are only received after 15 days of absence of work, and that the data for the USA refer to cases of 1 day or more of absenteeism, it appears that the fivefold difference will be much larger with comparable eligibility criteria for a sickness absence benefit.

In recent years, several authors have pointed out that multisite pain can represent a much higher impact on daily life of workers and on society than a musculoskeletal complaint affecting solely one body site. Recent studies have presented evidence that multisite musculoskeletal pain (MMP) is more often present than single-site musculoskeletal pain (Parot-Schinkel et al. 2012; Miranda et al. 2010; Kamaleri et al. 2008; Fernandes et al. 2016). However, less is known about consequences of this musculoskeletal multimorbidity, in terms of healthcare utilization and loss of productivity due to sickness absence and restricted work.

Despite the apparent importance of multisite musculoskeletal pain for consequences in terms of performance at work and healthcare utilization, most studies have focused on single outcome measures (Andersen et al. 2011; Matsudaira et al. 2011). Several studies have reported associations of multisite pain with general sickness absence rather than sickness absence caused by musculoskeletal diseases. Even though cross-studies comparisons can be difficult due to differences between self-reported and registered sickness absence and temporary or long-term sickness absence, these studies consistently demonstrate that the number of pain sites is an important and independent risk factor (Haukka et al. 2013; Kamaleri et al. 2009). Nyman et al. (2007), in a study about sickness absence and concurrent low back and neck–shoulder pain, reported that comorbidity was associated with both short-term and long-term total sickness absence. Other studies have shown that multisite pain is a risk factor for reduced work ability and poor functioning (Neupane et al. 2011; Miranda et al. 2010; Kamaleri et al. 2008; Saastamoinen et al. 2006).

There is still a lack of studies that have investigated the influence of multisite musculoskeletal pain on functioning at work, sickness absence and healthcare utilization, due to musculoskeletal diseases. IJzelenberg and Burdorf (2004) found mixed evidence among industrial workers with low back pain: In spite of subjects with high pain intensity or disabling low back pain being more likely to have musculoskeletal comorbidity of the neck and upper extremities, no impact of upper extremity comorbidity was found on healthcare utilization and sickness absence for low back pain. Conversely, Neupane et al. (2015), found an association between multisite pain and future sickness absence due to musculoskeletal diagnosis.

The aims of this study were (i) to describe the healthcare utilization, sickness absence and restrictions of work due to musculoskeletal pain in different body sites and according to number of pain sites and (ii) to investigate differences in associations between multisite and single pain with healthcare utilization, sickness absence and restrictions at work in workers from Bahia, Brazil.

## Methods

### Study population and setting

This study was based on two cross-sectional surveys carried out in 2010 and 2012. The study population comprised workers from shoe industry companies and workers from urban cleaning services in the state of Bahia, Brazil. The response in the pooled data was 97 % (*n* = 1070, 228 women and 842 men). In two shoe manufacturers, a stratified random sample was taken, proportional to the number of employees in each company and proportional to gender. The second population consisted of all urban cleaning workers, the maintenance and operation staff from the company that provides service to Salvador City, Bahia, Brazil. All participants were employed at the moment of the study. In the shoe industry, the main occupations involved were assemblers or manufacturers of shoes and machine operators, and occupations in urban cleaning services were garbage collectors, truck drivers and maintenance workers.

To collect the data, interviews with a structured questionnaire were conducted, by a team of trained interviewers consisting of one health and safety engineer, three physiotherapists, one ergonomist and four academics from the physiotherapy course. They were aware about being sure of clarifying all questions.

Data were collected at each participating company, during a regular working day, in a reserved place, ensuring privacy to workers. The Research Ethics Committee of Hospital São Rafael and of Escola de Enfermagem of Universidade Federal da Bahia approved the study proposals. Each worker signed an informed consent form before answering the questions during the interview. Data collection was preceded by meetings with researchers and workers inside each company. Detailed information about these meetings is described elsewhere (Fernandes et al. 2016).

### Dependent variables

During the interviews, a structured questionnaire was used with questions on three different outcomes of this study: healthcare utilization, sickness absence and restricted work due to musculoskeletal pain. Presence of each one of the outcomes was measured by questions, whose phrases were: 1. Have you had medical treatment for the problem? 2. How many days of work lost in the last year due to the problem? 3. How many days of light or restricted duty in the last year due to the problem? Responses on questions 2 and 3 were dichotomized in Yes, for one or more days, and No, for zero days of sickness absence or restricted work. The phrasing of the questions ensured that Brazilian workers could distinguish between sickness absence and restricted duties in the current job. The latter is instigated by an occupational physician or supervisor and may be seen as a temporary measure of adaptations at work for workers with limiting health problems. Since for multisite pain it was deemed too difficult to differentiate between these outcome measures for a particular body site, healthcare utilization, sickness absence and restricted duty were determined across all body sites rather than for each musculoskeletal pain separately.

### Independent variables

The main independent variable, presence of musculoskeletal pain in the previous 12 months, was investigated for the following body sites: upper limbs (hand, wrist, forearm, elbow), neck, shoulder, upper back, lower back, upper legs, knees, lower legs, ankles and feet, using the enlarged version of the Nordic Musculoskeletal Questionnaire (Kuorinka and Forcier 1995).

The number of pain sites is the sum score of all body sites with pain, then collapsed in four strata: 0 = single-site pain; 1 = two-sites pain; 2 = three-sites pain; and 3 = four or more sites of pain.

The questionnaire included the covariates: age, gender, physical and psychosocial work demands and leisure-time physical activities. Direct measurements of weight and height followed the interviews.

Workers’ self-reports on a set of questions using a six-point scale, ranging from 0 to 5 (scale of duration), with verbal qualifiers at the ends (0 = ”never” and 5 = ”all the time”), were used to measure physical work demands. Questions were asked about repetitive hand movements; general working postures like sitting, standing, walking; awkward postures like arms above shoulder height, trunk bent forward or trunk twisted, and squatting; material handling like weight lifting, pulling or pushing; and mechanical grip force on the object of work. For this last variable, we used a six-point response scale on intensity and verbal qualifiers at the ends were “too weak” = 0 and “too strong” = 5.

A version of the Job Content Questionnaire (JCQ), translated into Portuguese and validated by Araújo and Karasek (2008), was used to measure psychosocial work demands. Cronbach’s alpha coefficients of this version revealed acceptable internal consistency, and factor analysis showed high consistency with the theoretical model (Karasek 1979). Job demands were measured by nine questions. Job control was measured by nine questions, six of them related to skills and three questions on authority to make decisions. Sum scores across job control and job demands were dichotomized at median scores to define high strain (high demand and low control). For social support, one variable, dichotomized by median scores, combined support from co-workers and support from supervisors.

Leisure-time physical activities (LTPA) were measured by means of a question about what the worker mainly does while not working in the company or at home, with a four-item response scale: 1. competitive sports activity, 2. running, doing gymnastics, swimming, playing football and bike riding, 3. walking, fishing and gardening, 4. reading the newspaper or a magazine, watching television and studying. Individuals with answers 1, 2 or 3 were considered as active in leisure-time.

Body mass index (BMI, kg/m^2^) was calculated based on direct measurements of height and weight: low weight <18.5, normal 18.5–25, overweight ≥25–30 and obesity ≥30 kg/m^2^. A dichotomized variable was used: normal <25 or overweight/obesity ≥25.

### Statistical analysis

First, the presence of pain in eight different body sites in the past 12 months with proportions of healthcare utilization, sickness absence and restricted work was described.

In order to reduce the number of variables on physical load and to prevent variable redundancies, a factor analysis was performed and captured the nine variables of physical exposure into two latent factors that explained 63 % of the total variance among workers in the study population. The initial extraction was made through the main components of the model, and factors were obtained without rotation. Their composition, in a decreasing order of the loads presented by each variable, was as follows: Factor 1 (initial eigenvalue = 4.494; variance = 49.9 %) characterized physical demands of material handling and strenuous postures including awkward postures: pulling, lifting and pushing weights, squatting, arms above shoulder height, trunk bending, trunk rotation and mechanical hand pressure on the object of work. Factor 2 (initial eigenvalue = 1.186; variance = 13.2 %) characterized repetitive work with the variable “repetitive hand movements.” Both factors were used as independent variables for physical work demands, with cutoff points at median values.

The associations between number of pain sites and healthcare utilization, sickness absence and restricted work were assessed by means of Cox regression, presenting the prevalence ratio (PR) and 95 % confidence interval. For the purpose of analyses, we restricted our sample to those respondents who had pain (*n* = 869), and individuals with a single-site pain were the reference group.

Two different Cox regression models to each one of the three dependent variables determined the associations between number of pain sites with the outcome, i.e., healthcare utilization, sickness absence and restricted work. In the first model, associations were adjusted for age, sex, BMI and LTPA. In the second model, additional adjustment was obtained for job strain (psychological demand and job control), social support, manual material handling and awkward postures (Factor 1) and repetitive movements (Factor 2).

In studies with an outcome measure with high prevalence, such as musculoskeletal pain, the odds ratio cannot be interpreted as a risk ratio as it will severely overestimate the associations. So, we conducted Cox regression analysis, based on Coutinho et al. (2008), in order to provide prevalence ratio estimates. According to these authors, the comparative analyses of cross-sectional studies showed that the Cox and Poisson models with robust variance are better alternatives than logistic regression analysis. They advise that “The Cox regression model is usually used to analyze time-to-event data. In cross-sectional studies, no time periods are observed, but if a constant risk period is assigned to all the individuals in the study, the hazard ratio estimated using Cox regression equals the PR”.

Since our total study population is not a random sample of the Brazilian workforce or specific occupational groups therein, the inferential statistics presented serves merely “as a minimum estimate of the actual uncertainty about the object of estimation” (Rothman et al. 2008).

All descriptive and inferential statistical analyses were performed using the SPSS software, version 21.

## Results

Among 1070 workers, 869 (81.2 %) reported any musculoskeletal pain in the previous 12 months. Around 44 % of workers reported low-back pain, and the second highest prevalent pain was in upper limbs, reported by 37 % of workers. Less workers reported ankle or feet pain. Around 38 % of those with pain sought healthcare services due to musculoskeletal pain. Much less workers left work due to pain (12.1 %) and only 6.8 % stayed in restricted work because of musculoskeletal symptoms. Proportions of each consequence, according to the body site of pain, did not show great differences, although workers with low back pain consistently showed a higher proportion of healthcare utilization, sickness absence and restricted work (Table 1).

**Table 1:** Proportion of healthcare utilization, sickness absence and restricted work, according to pain in the previous 12 months in eight body sites, among 1070 workers in Brazil

Body sites of pain	*n*	Prevalence (%)	Consequences within those workers with musculoskeletal pain					
			Healthcare utilization		Sickness absence		Restricted work	
			*n*	%	*n*	%	*n*	%
Neck	235	22.0	57	24.2	38	16.2	21	8.9
Shoulder	275	25.7	61	22.2	37	13.4	24	9.8
Upper back	282	26.4	67	23.8	32	11.3	11	3.9
Upper limbs	398	37.2	86	21.6	47	11.8	40	10.0
Low back	466	43.6	130	27.9	85	18.2	54	11.6
Upper leg/knee	282	26.4	78	27.7	42	14.9	32	11.3
Lower leg	290	27.1	68	23.4	32	11.0	19	6.5
Ankle/feet	185	17.3	44	23.8	21	11.3	13	7.0
Any body site	869	81.2	329	37.9	105	12.1	59	6.8

From 1070 workers, 24.3 % had a single-site pain. It represents around 30 % of people with pain (260/869). The majority of workers had pain in more than one body site (57 %). Among workers with pain, with increasing number of pain sites the occurrence of healthcare utilization, sickness absence and restricted work also increased. Among those with a single-site pain, 28.5 % sought medical treatment while this proportion raised to 47 % among people with four or more sites with pain. In addition, sickness absence ranged from 6.5 % up to 20.5 % and restricted work, from 3.1 % up to 9.4 %. See Table 2.

**Table 2:** Healthcare utilization, sickness absence and restricted work due to musculoskeletal complaints, according to number of pain sites among 1070 workers in Brazil

Number of pain sites	*n*	Healthcare utilization (%)*	Sickness absence (%)*	Restricted work (%)**
No pain	201	–	–	–
Single site	260	28.5	6.5	3.1
Two sites	218	35.8	10.6	6.4
Three sites	157	43.9	10.8	9.6
Four or more sites	234	47.2	20.5	9.4

Chi-square for trend, * *p*2 = 0.000, ** *p*2 = 0.018

**Fig. 1:**
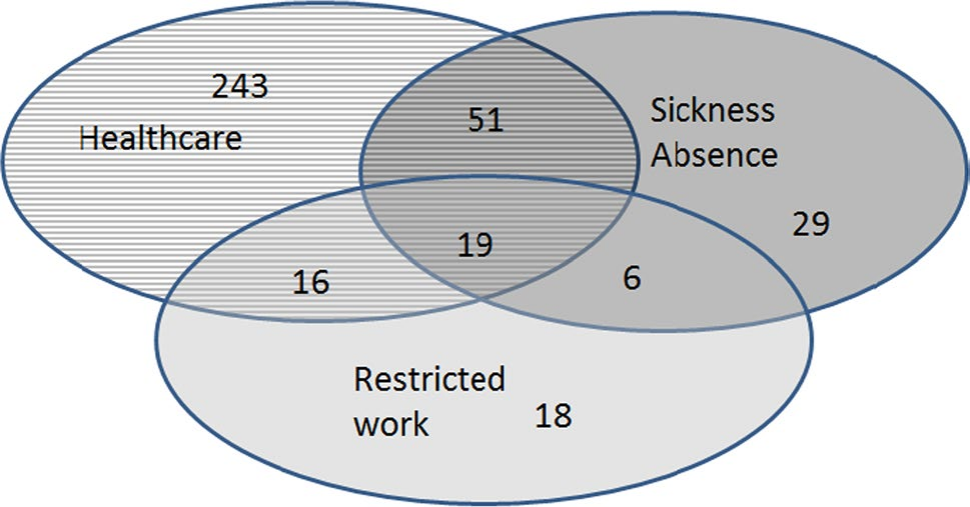
Healthcare utilization, sickness absence, restricted work and their intersections, among workers with one or more consequences due to musculoskeletal pain

Figure 1 shows the considerable overlap between the three consequences of musculoskeletal pain. Among 869 workers with musculoskeletal pain, 44 % (382) had one or more consequences, like seeking health care or having sickness absence or staying in restricted work due to the pain. Among those who sought a healthcare attendance in the previous 12 months (*n* = 329), 15.5 % (51) had also sickness absence, 5.8 % (19) reported restricted work and sickness absence, and 4.9 % (16) reported restriction at work. Sickness absence and restrictions at work were strongly associated, whereby among workers with restrictions at work, also 42 % reported sickness absence and among those with sickness absence, 24 % reported restricted work.

Table 3 shows that in the first model, adjusted for age, gender, BMI and LTPA, having pain in two sites increased the prevalence of healthcare utilization by 30 % compared with having a single-site pain. For individuals with pain in four or more body sites, the utilization of health care was 1.6-fold the utilization by people with a single-site pain. Additional adjustment for occupational demands (psychosocial and physical demands) varied only slightly the association between healthcare utilization and number of pain sites. Having pain in four or more sites increased the prevalence of sickness absence threefold compared with people with single-site pain. After adjustment by occupational demands, this association increased to 3.6. The same can be seen for prevalence of restricted work: additional adjustment by occupational demands increased the prevalence ratio from 3.5 to 4.0 among workers with four or more sites with pain compared with those with single-site pain (Table 3).

**Table 3:** Association between healthcare utilization, sickness absence and restricted work with number of pain sites

Number of pain sites	Healthcare utilization		Sickness absence		Restricted work	
	PR 95 % CI^a^	PR 95 % CI^a,b^	PR 95 % CI^a^	PR 95 % CI^b^	PR 95 % CI^a^	PR 95 % CI^b^
Single site	1	1	1	1	1	1
Two sites	1.3 (0.9–1.8)	1.3 (1.0–1.9)	1.6 (0.8–3.0)	2.1 (1.0–4.1)	2.1 (0.8–5.0)	2.4 (0.9–6.0)
Three sites	1.5 (1.1–2.2)	1.6 (1.1–2.3)	1.6 (0.8–3.1)	2.0 (0.9–4.0)	3.3 (1.4–7.8)	3.6 (1.4–8.9)
Four or more sites	1.6 (1.2–2.2)	1.7 (1.2–2.4)	3.0 (1.7–5.2)	3.6 (2.0–6.8)	3.5 (1.5–8.0)	4.0 (1.7–9.5)

^a^Model 1: adjusted for age, gender

^b^Model 2: additional adjustment for job strain, social support, manual material handling and awkward postures, repetitive movements

## Discussion

In this study with 1070 workers actively engaged with daily tasks at work, we found a high prevalence of musculoskeletal pain in the previous 12 months (81.2 %), and around two-thirds of these workers had pain in more than one body site. Around 38 % of the workers with musculoskeletal pain sought healthcare advice for their pain. Workers with pain also reported sickness absence (12 %) and restricted work (7 %). It is noteworthy that these consequences are not equally distributed in the sample. We found consistently an increasing occurrence of healthcare utilization, sickness absence and restricted work with increasing number of pain sites.

Our high prevalence of musculoskeletal pain compares well with the study by Parot-Schinkel et al. (2012), who found in a working population in France a 12-month prevalence of 83.8 % and two-thirds of workers reported pain in more than one body site. It may be argued that a recall period of 12 months cannot differentiate between concurrent or consecutive periods of musculoskeletal pain. In an additional analysis in our study population, we observed comparable musculoskeletal comorbidity for pain in the past 7 days, for example, among those with low back pain 72 % reported another musculoskeletal pain. This finding indicates that the observed comorbidity reflects primarily widespread pain across different body regions (Fernandes et al. 2016). This high comorbidity is in accordance with findings by Kamaleri et al. (2008), who also assessed multisite pain in the previous 7 days.

In this study, we were interested in associations of multisite pain with healthcare utilization and functioning at work. Since these consequences of musculoskeletal pain are less frequent, we had to focus on 12-month pain, instead of pain in previous 7 days. Descriptions of pain according to different body sites will help us to capture the most affected body sites, but these descriptions should be necessarily supplemented by information about the comorbidity. For instance, even if low back pain is reported by 40 % of people (Table 1), the great amount of these people has pain in at least one other body site.

Proportions of healthcare utilization, sickness absence and restricted work did not vary substantially according to eight different body sites, although workers with low back pain showed a slightly higher proportion of these consequences. Even though these proportions of consequences according to pain locations indicate by themselves a burden of loss of productivity and impact on health-related quality of life, the number of pain sites seems to be more important to determine the consequences than the body site where the pain occurs. This may be related to the high prevalence of comorbidity that hampers us to clearly link each kind of consequence with a specific body site of pain. Nonetheless, in this cross-sectional survey with only a minority of workers without musculoskeletal comorbidity, we cannot reject the possibility that workers with low back pain are at higher risk for healthcare utilization, sickness absence and restricted work than workers with musculoskeletal pain in other body regions. It would be of interest to be able to determine whether workers with low back pain in physically demanding jobs, such as shoe industry and cleaning services, have higher healthcare utilization and sickness absence than workers with low back pain in less strenuous jobs.

Saastamoinen et al. (2006) investigated location of pain, number of painful locations and chronicity of pain and found that “the number of painful locations was associated with large variation in functioning, whereas the locations themselves were less important to the variation in functioning.” In accordance with these findings, we have consistently found an increasing trend of healthcare utilization with increase in number of pain sites, which is 28.5 % among those with single-site pain, rising to 47 % among people with four or more sites with pain. This increasing trend was also observed for the number of pain sites with sickness absence and restricted work. These findings clearly suggest that the functional consequences of pain depend on how much body regions are affected, i.e., the more widespread pain, the higher the likelihood of medical consumption, sickness absence and restricted work.

Despite the scarce literature about the impact of the number of pain sites on healthcare utilization or on restricted work, results by some authors are in line with the importance of number of painful sites for functioning at work and health-related functioning. Neupane et al. (2011), Miranda et al. (2010) and Kamaleri et al. (2008) found that poorer work ability or lower functioning, in working population, was consistently related to the number of painful areas. Kamaleri et al. (2008) state “for all functional ability scales, there was an almost linear increase in functional problems with increasing numbers of pain sites”.

A few studies have also reported associations between musculoskeletal comorbidity and sickness absence or associated measures. Our findings are compatible with those by Kamaleri et al. (2009), who found that number of pain sites predicted presence of a disability benefit. Haukka et al. (2013) assessed the association between number of pain sites and sickness absence trajectories, based on the national registries of the Social Insurance Institution of Finland, and found that the number of pain sites was a strong and independent predictor of general sickness absence. Studies on musculoskeletal sickness absence have corroborated the importance of multisite pain. Neupane et al. (2015) found that multisite pain predicted sickness absence due to diagnosis of musculoskeletal disorders, based on data from the personnel register of a food industry company. In addition, Morken et al. (2003) found that widespread pain was a predictor of sickness absence due to musculoskeletal disorders among workers in the aluminum industry.

 The most frequent consequence among people with pain was to seek healthcare service (38 %) in order to relieve their symptoms. Getting a sickness absence happened much less (12 %) than seeking health care, as expected, but was more prevalent than continuing to work with restrictions (7 %). This finding raises an important point about possible barriers for workers with musculoskeletal complaints. Instead of having the possibility for adaptation of their work demands in order to allow them to continue performing, it was more frequent that workers got a sickness absence. The primary prevention of pain in workplaces has to be seen as the main challenge for companies in our study population, but given the high prevalence of musculoskeletal complaints, promoting accommodations of workplace for workers already affected by pain, in order to decrease the necessity of sickness absence, also seems to be an important prevention strategy. In accordance with National Research Council and the Institute of Medicine (2001), secondary prevention, which is undertaken after individuals have experienced pain, requires introduction of job redesign. Even for chronically disabled workers, it is possible to provide working conditions that will enable them to keep working, within the limitations of the individual’s impairments, by means of tertiary prevention strategies, which “are usually made on a case by case basis.” Hence, considering that musculoskeletal disorders and consequent work disability are potentially preventable, and given the importance of multisite pain for reduced health among workers, the number of pain sites seems to be a useful measure in order to anticipate interventions at workplaces for prevention of pain and for decreasing the number of pain sites. In this way, instead of focusing on prevention of a single-site pain, the prevention programs should address physical and psychosocial demands at work, in view of allowing workers to perform their tasks under suitable conditions to the whole body instead of one particular body region. Besides, interventions aiming to prevent musculoskeletal pain in workplaces, guided by epidemiological evidence on its risk factors, shall incorporate the participation of workers in job redesign, bringing their daily experience in facing the demands at work, in order to enlarge the effectiveness of the adopted improvements.

### Methodological considerations

The study was carried out inside workplaces, characterizing our population as a working population, instead of a general population. In spite of its cross-sectional design, strategies adopted in order to minimize information bias and selection bias, also including information about possible confounders, seem to strengthen our study. We ensured absolute privacy and confidentiality, and information was collected by independent researchers from the public and respectful institution that is Federal University of Bahia. No participation of employers was allowed in the course of the study, in order to avoid conflicting interests that would bias the results. Besides, doing the data collection by means of interviewer-administered questionnaire of a high-qualified team certainly contributed to the high response, also among those workers with modest or little reading and writing skills.

We used self-reports on healthcare utilization, sickness absence and restricted work. Some studies on multisite pain and sickness absence have used register data from social security benefits systems. These registers are usually regarded to present more objective data, but studies based on self-reports about sickness absence have shown consistent and comparable results. On the other hand, self-reports about sickness absence, although more prone to recall bias, will protect the privacy of workers and, thus, will facilitate participation in studies.

We also used self-reports for assessing the presence of musculoskeletal complaints, and psychosocial and physical work demands, which could represent a weakness of the study. Nevertheless, self-reports have been assumed to be the best way of assessing pain, given the subjective nature of this health outcome. A similar reason can be assumed for psychosocial demands, measured by means of a validated version of Job Content Questionnaire. Besides, physical demands were measured by means of a wide set of questions on general body postures, on postures of specific body sites, on material handling and on repetitive movements. A strength of our approach was the use of a factor analysis, which captured the variance of total physical load into two factors instead of using nine different variables. This approach seems to be more valid presentation of complex patterns of physical load, especially when compared to the use of single-item questions for assessing physical exposure at work, as seen in the literature. As for the common critique on self-reported physical demands, Stock et al. (2005), in a systematic review, have pointed out many issues about considering uncritically the direct measurements or observational methods as gold standard in assessing physical demands at work. Therefore, after considering the advantages and disadvantages of methods to accurately measure physical demands at work, we feel that our approach supports that questionnaires still play an essential role in the assessment of physical demands at work in epidemiological studies (Burdorf and van der BeeK 1999a, b). In this study with 1070 workers, we were able to apply, by means of interview, a structured set of questions that allowed us to reasonably capture the variability of exposure, in a work context full of diversity. This would be hard to achieve with observational methods or direct measurements, as their use requires a large measurement effort and subsequent resources which makes data collection at individual level almost impossible (Stock et al. 2005). The choice of the questions and response scales considered that they were meaningful to the study population, workers from cleaning services and from shoe manufacturing. To apply questionnaire by means of interview has assured the possibility of clarifying any item when necessary, and certainly improved the quality of workers’ responses.

In summary, this study showed that: 1. Multisite pain accounted for two-thirds of musculoskeletal pain in the previous 12 months; 2. there is a consistently increasing occurrence of healthcare utilization, sickness absence and restricted work with increasing number of pain sites; 3. the functional consequences of pain depend on how much body regions are affected, i.e., the more widespread pain, the higher the likelihood of medical consumption, sickness absence and restricted work; 4. given the high comorbidity, the number of pain sites, instead of specific body site of pain, seems to be a useful measure for musculoskeletal complaints and a target for prevention strategies.

## References

[CR1] Andersen LL, Mortensen OS, Hansen JV, Burr H (2011). A prospective cohort study on severe pain as a risk factor for long-term sickness absence in blue- and white-collar workers. Occup Environ Med.

[CR2] Araújo TM, Karasek R (2008) Validity and reliability of the job content questionnaire in formal and informal jobs in Brazil. Scand J Work Environ Health 0(suppl 6):52–59

[CR3] Brasil. Ministério da Previdência Social (2014) Anuário Estatístico da Previdência social 2012. Acidentes de trabalho registrados segundo CID. Disponível em: http://www.mpas.gov.br. Acesso em 19 de abril de 2014

[CR4] Burdorf A, van der BeeK A (1999) Exposure assessment strategies for work-related risk factors for musculoskeletal disorders. Scand J Work Environ Health 25(suppl 4):25–30 10628437

[CR5] Burdorf A, van der BeeK A (1999). In musculoskeletal epidemiology are we asking the unanswerable in questionnaires on physical load?. Scand J Work Environ Health.

[CR6] Coutinho LMS, Scazufca M, Menezes PR (2008). Methods for estimating prevalence ratios in cross sectional studies. Rev Saúde Pública.

[CR7] Fernandes RCP, Pataro SMS, de Carvalho RB, Burdorf A (2016) The concurrence of musculoskeletal pain and associated work-related factors: a cross sectional study with Brazilian workers. Rotterdam, Erasmus MC, working paper

[CR8] Haukka E, Kaila-Kangas L, Ojajärvi A, Miranda H, Karppinen J, Viikari-Juntura E, Heliövaara M, Leino-Arjas P (2013). Pain in multiple sites and sickness absence trajectories: a prospective study among Finns. Pain.

[CR9] Ijzelenberg W, Burdorf A (2004) Impact of musculoskeletal co-morbidity of neck and upper extremities on healthcare utilisation and sickness absence for low back pain. Occup Environ Med 61(10):806–810 10.1136/oem.2003.011635 PMC1740669 15377765

[CR10] Kamaleri Y, Natvig B, Ihlebaek CM, Bruusgaard D (2008). Localized or widespread musculoskeletal pain: does it matter?. Pain.

[CR11] Kamaleri Y, Natvig B, Ihlebaek CM, Bruusgaard D (2009). Does the number of musculoskeletal pain sites predict work disability? A 14-year prospective study. Eur J Pain.

[CR12] Karasek R (1979). Job demand, job decision latitude, and mental strain: implications for job redesign. Adm Sci Q.

[CR13] Kuorinka I, Forcier L (1995). Work related musculoskeletal disorders (WMSDs): a reference book for prevention.

[CR14] Matsudaira K, Palmer KT, Reading I, Hirai M, Yoshimura N, Coggon D (2011). Prevalence and correlates of regional pain and associated disability in Japanese workers. Occup Environ Med.

[CR15] Miranda H, Kaila-Kangas L, Heliovaara M, Leino-Arjas P, Haukka E, Liira J, Viikari-Juntura E (2010). Musculoskeletal pain at multiple sites and its effects on work ability in a general working population. Occup Environ Med.

[CR16] Morken T, Riise T, Moen B, Hauge SHV, Holien S, Langedrag A, Pedersen S, Saue ILL, Seljebø GM, Thoppil VT (2003). Low back pain and widespread pain predict sickness absence among industrial workers. BMC Musculoskeletal Disorders.

[CR17] National Research Council and the Institute of Medicine (2001) Musculoskeletal disorders and the workplace: low back and upper extremities. Panel on musculoskeletal disorders and the workplace. Commission on behavioral and social sciences and education. National Academy Press, Washington, DC

[CR18] Neupane S, Miranda H, Virtanen P, Siukola A, Nygard C-H (2011). Multi-site pain and work ability among industrial population. Occup Med.

[CR19] Neupane S, Leino-Arjas P, Nygard C-H, Miranda H, Siukola A, Virtanen P (2015). Does the association between musculoskeletal pain and sickness absence due to musculoskeletal diagnoses depend on biomechanical working conditions?. Int Arch Occup Environ Health.

[CR20] Nyman T, Grooten WJA, Wiktorin C, Liwing J, Norrman L (2007). Sickness absence and concurrent low back and neck-shoulder pain: results from the MUSIC—Norrtalje study. Eur Spine J.

[CR21] Parot-Schinkel E, Descatha A, Ha C, Petit A, Leclerc A, Roquelaure Y (2012). Prevalence of multisite musculoskeletal symptoms: a French cross-sectional working population-based study. BMC Musculoskeletal Disorders.

[CR22] Rothman KJ, Greenland S, Lash TL (2008). Precision and statistics in epidemiologic studies. Modern epidemiology.

[CR23] Saastamoinen P, Leino-Arjas P, Laaksonen M, Martikainen P, Lahelma E (2006) Pain and health related functioning among employees. J Epidemiol Commun Health 60:793–798 10.1136/jech.2005.043976 PMC2566029 16905725

[CR24] Souza NSS, Santana VS (2011) Cumulative annual incidence of disabling work-related musculoskeletal disorders in an urban area of Brazil. Cad. Saúde Pública, Rio de Janeiro 27(11):2124–2134 10.1590/s0102-311x2011001100006 22124490

[CR25] Stock S, Fernandes R, Delisle A, Vèzina N (2005). Reliability and validity of workers’ self-reports of physical work demands. Scand J Work Environ Health.

